# Supplementary material for: TBC1D9 regulates TBK1 activation through Ca2+ signaling in selective autophagy
Source: Nat Commun. 2020 Feb 7;11:770. doi: 10.1038/s41467-020-14533-4 (PMC7005872; doi:10.1038/s41467-020-14533-4)
Supplement: Supplementary file 1 — Supplementary Information [file 41467_2020_14533_MOESM1_ESM.pdf]

Cover Page – Supplementary Information

TBC1D9 regulates TBK1 activation through Ca^{2+} signaling in selective autophagy

Nozawa et al.

Supplementary Figure 1

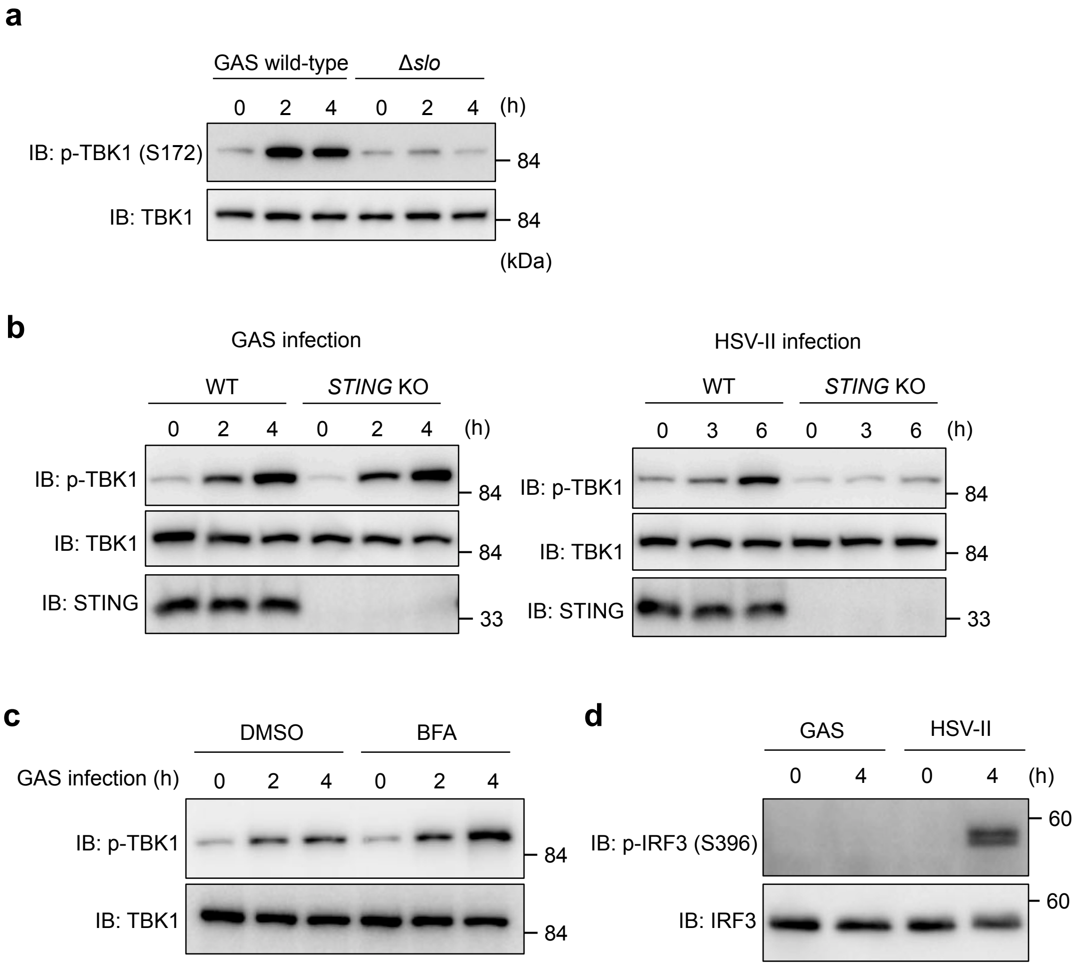

Supplementary Figure 1 GAS infection induces STING-independent TBK1 activation through SLO-dependent mechanism.

a Analysis of TBK1 activation during GAS infection. HeLa cells were infected with GAS wild-type (WT) or Δ slo mutant for indicated times, phosphorylated TBK1 at S172 and total TBK1 were detected by immunoblotting using specific antibodies. **b** TBK1 activation in *STING*-KO cells during GAS or HSV-II infection. **c** HeLa cells treated with brefeldin A (BFA) or DMSO for 1 h were infected with WT GAS. **d** Activation of IRF3 was analyzed by detecting phosphorylation of IRF3 S396 in HeLa cells infected with GAS or HSV-II for 4 h.

Supplementary Figure 2

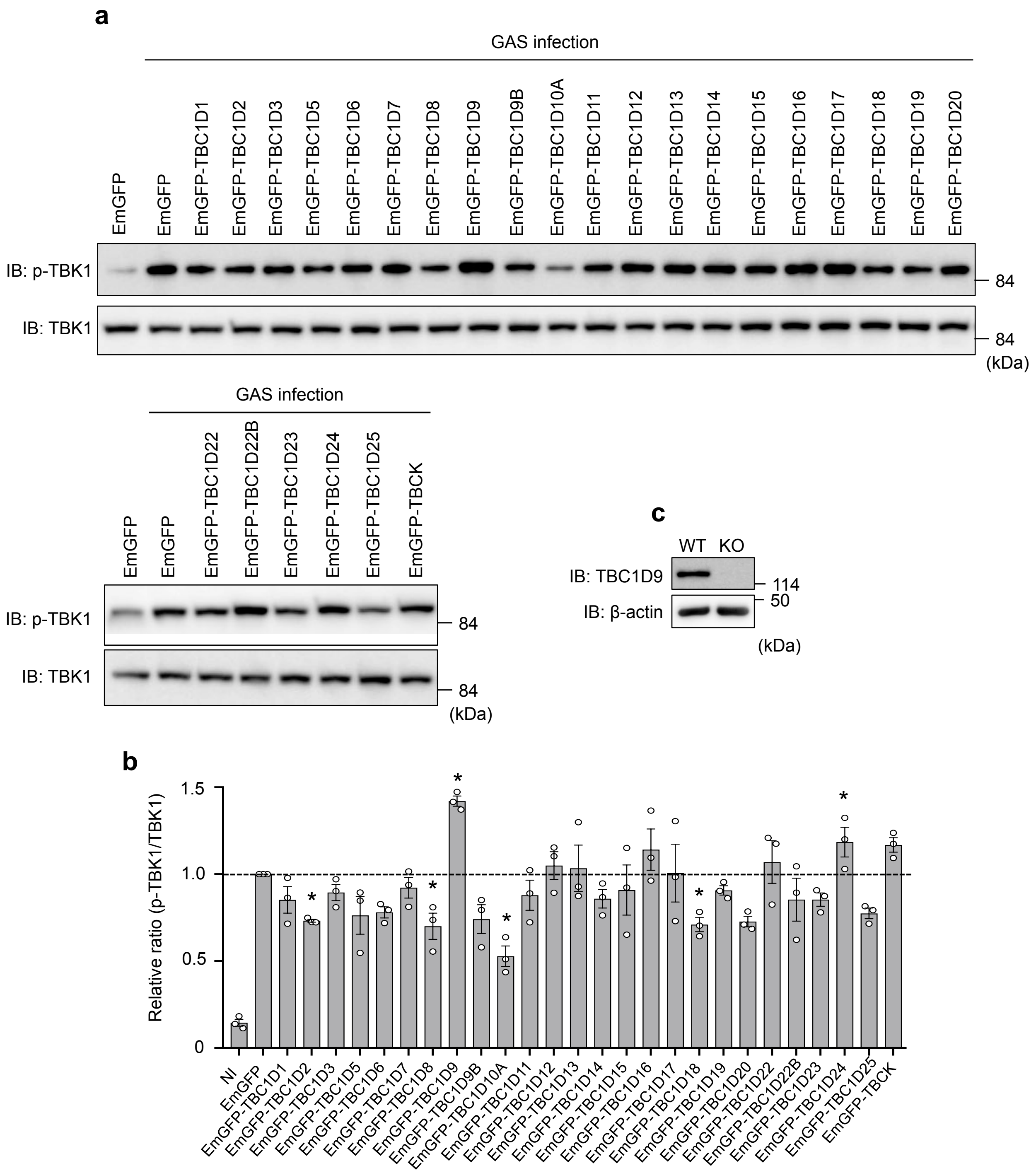

Supplementary Figure 2 Comprehensive screen of TBC/RabGAP proteins that affect on TBK1 activation during GAS infection.

a,b TBC/RabGAP screening involved in TBK1 activation during GAS infection. **a** Immunoblotting of phosphorylated-TBK1 (p-TBK1) and total TBK1 from EmGFP-TBC/RabGAP-expressing HeLa cells in response to GAS infection (4 h) and **b** quantification of TBK1 activation. Data represent the mean ± SEM of three independent experiments. **c** Immunoblotting of *TBC1D9*-KO cells. *P*-values calculated by two- tailed Student's t-test. ****p*-value < 0.001.

Supplementary Figure 3

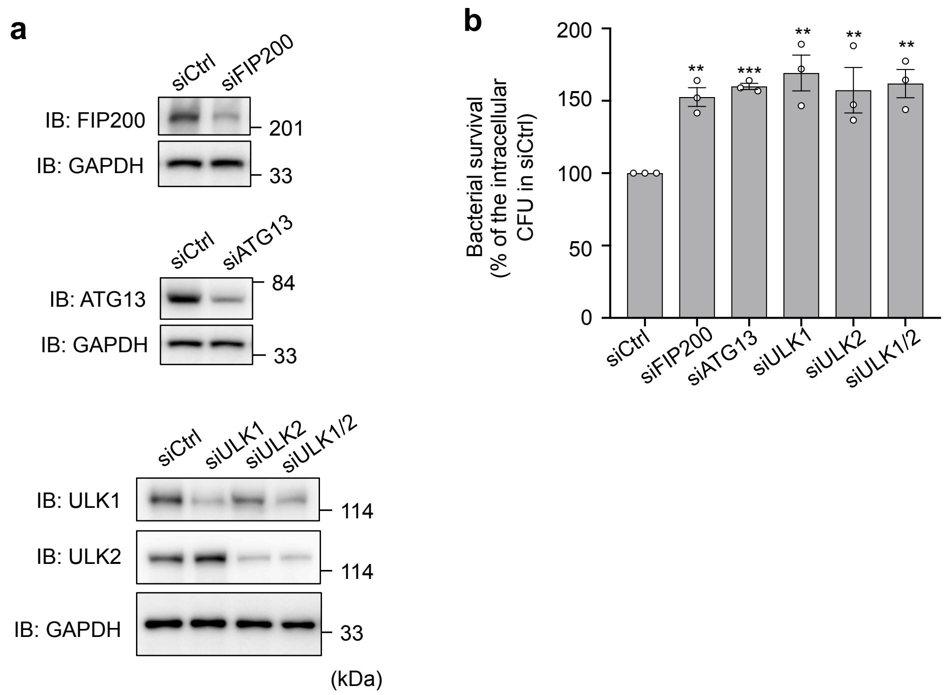

Supplementary Figure 3 ULK1 complex is required for xenophagic degradation of GAS.
a Immunoblotting of siRNA-transfected cells. HeLa cells were transfected with indicated siRNA oligonucleotides for 48 h, and cell lysate were analyzed by immunoblotting using specific antibodies.
b Intracellular bacterial CFU at 6 h post infection (hpi) in each siRNA-treated HeLa cells . Data represent the mean ± SEM of three independent experiments. *P*-values calculated by two- tailed Student's t-test. ***p*-value < 0.01, ****p*-value < 0.001.

Supplementary Figure 4

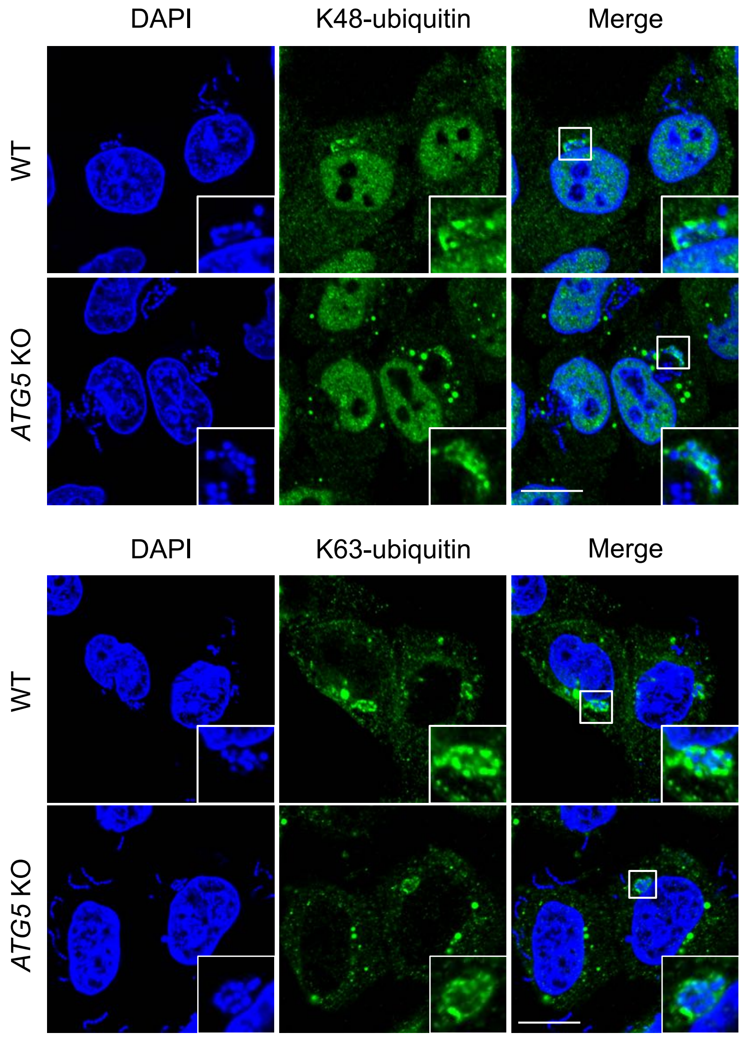

Supplementary Figure 4 Intracellular GAS is marked with K48 and K63 ubiquitin in an ATG5-independent manner,

HeLa WT and *ATG5*-KO cells were infected with GAS and fixed at 4 h, and stained for K48- or K63-ubiquitin using specific antibodies. Cellular and bacterial DNAs were stained with DAPI. Scale bars, 10 μm.

Supplementary Figure 5

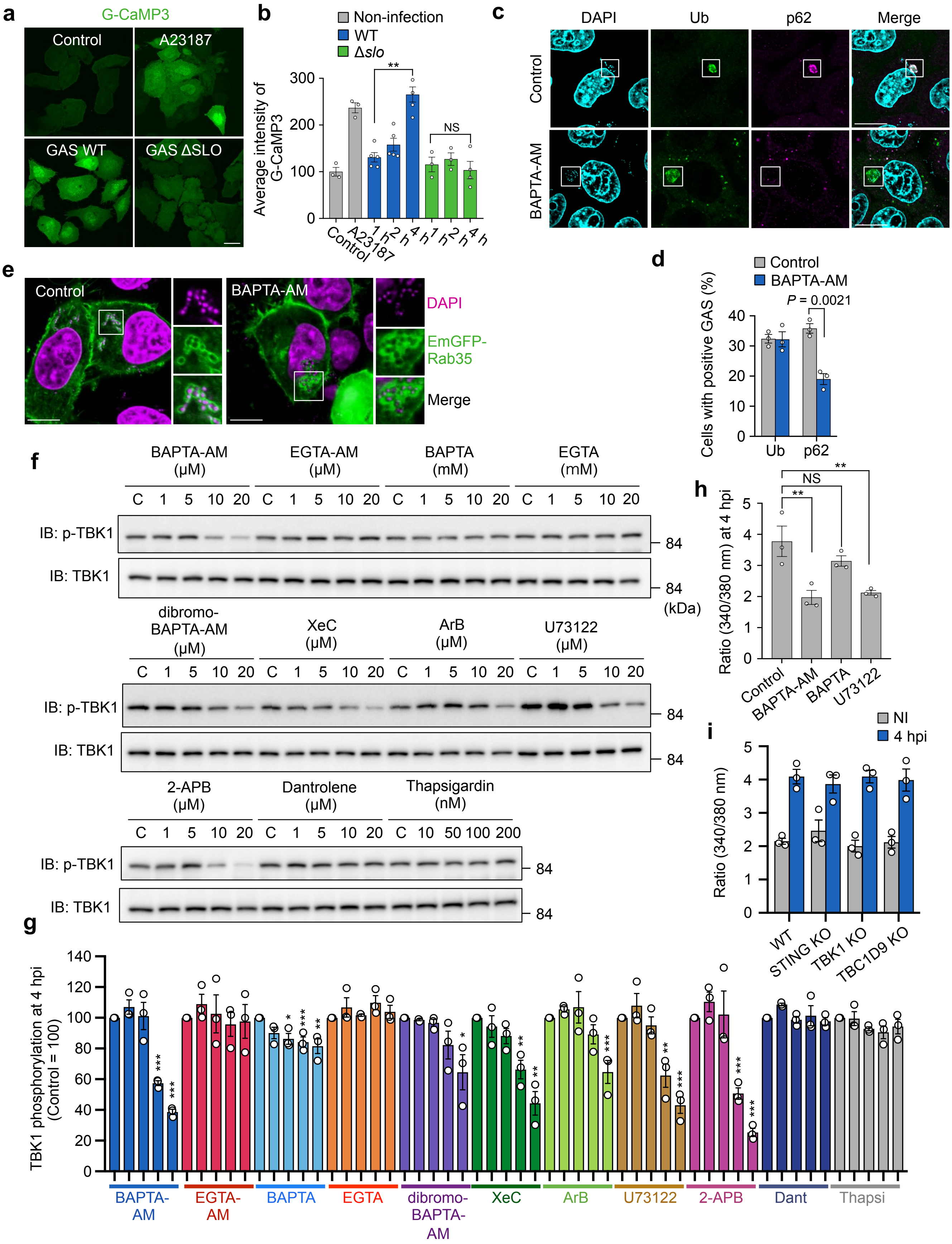

Supplementary Figure 5 Ca^{2+} signaling is critical for TBK1 activation in response to GAS invasion. **a,b** Intracellular Ca^{2+} mobilization during GAS infection. HeLa cells expressing the Ca^{2+} indicator G-CaMP3 were treated with A23187 or infected with GAS WT or Δ*slo* mutants. **a** Representative G-CaMP3-signal images and **b** quantification of G-CaMP3 intensity. **c,d** Effects of BAPTA-AM (10 μM) treatment on the recruitment of ubiquitin and p62 to GAS. HeLa cells treated with or without BAPTA-AM were infected with GAS for 4 h, fixed, and stained for poly-ubiquitin and p62. **c** Representative confocal images and **d** the percentage of infected-cells showing ubiquitin- or p62-positive GAS. **e** Representative confocal images of EmGFP-RAB35 recruitment to GAS in HeLa cells with or without BAPTA-AM (10 μM). **f,g** Effects of Ca^{2+} signaling inhibitors on TBK1 activation. **f** Immunoblotting of p-TBK1 and total TBK1 from GAS-infected HeLa cells treated with indicated inhibitors and **g** quantification of TBK1 activation. **h** Cytoplasmic Ca^{2+} elevation GAS-infected HeLa cells with indicated inhibitors. Ca^{2+} rise in response to infection was analyzed using Fura 2-AM. **i** Ca^{2+} dynamics in indicated KO cells during GAS infection. Scale bars, 10 μm. Data in **b**, **d, g, h,** and **i** represent the mean ± SEM of three independent experiments. *P*-values calculated by two- tailed Student's t-test. $^{*}p$-value < 0.05, $^{**}p$-value < 0.01, $^{***}p$-value < 0.001.

Supplementary Figure 6

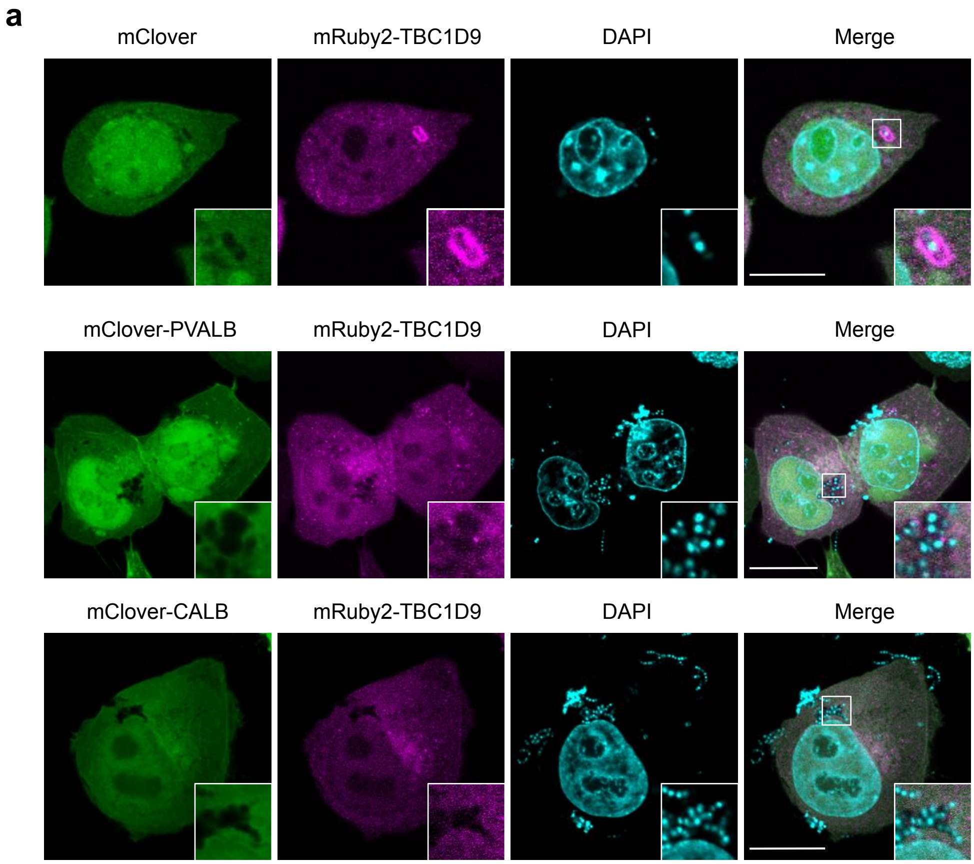

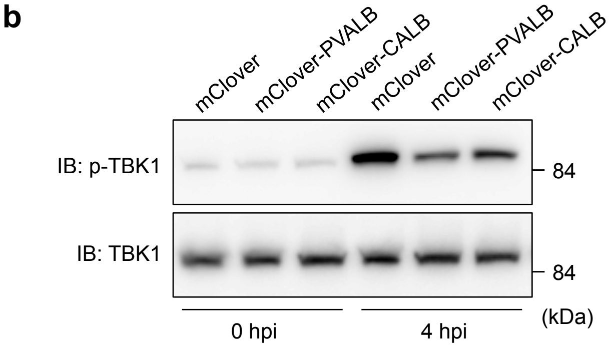

Supplementary Figure 6 Overexpression of Ca^{2+}-buffering protein inhibit TBC1D9 recruitment and TBK1 activation.

a HeLa cells transfected with mRuby2-TBC1D9 and either mClover, mClover-PCALB, or mClover-CALB were infected with GAS for 4 h. **b** HeLa cells transfected with mClover, mClover-PCALB, or mClover-CALB were infected with GAS for 4 h. The cell lysates were analyzed by immunoblotting using indicated antibodies. Scale bars, 10 µm.

Supplementary Figure 7

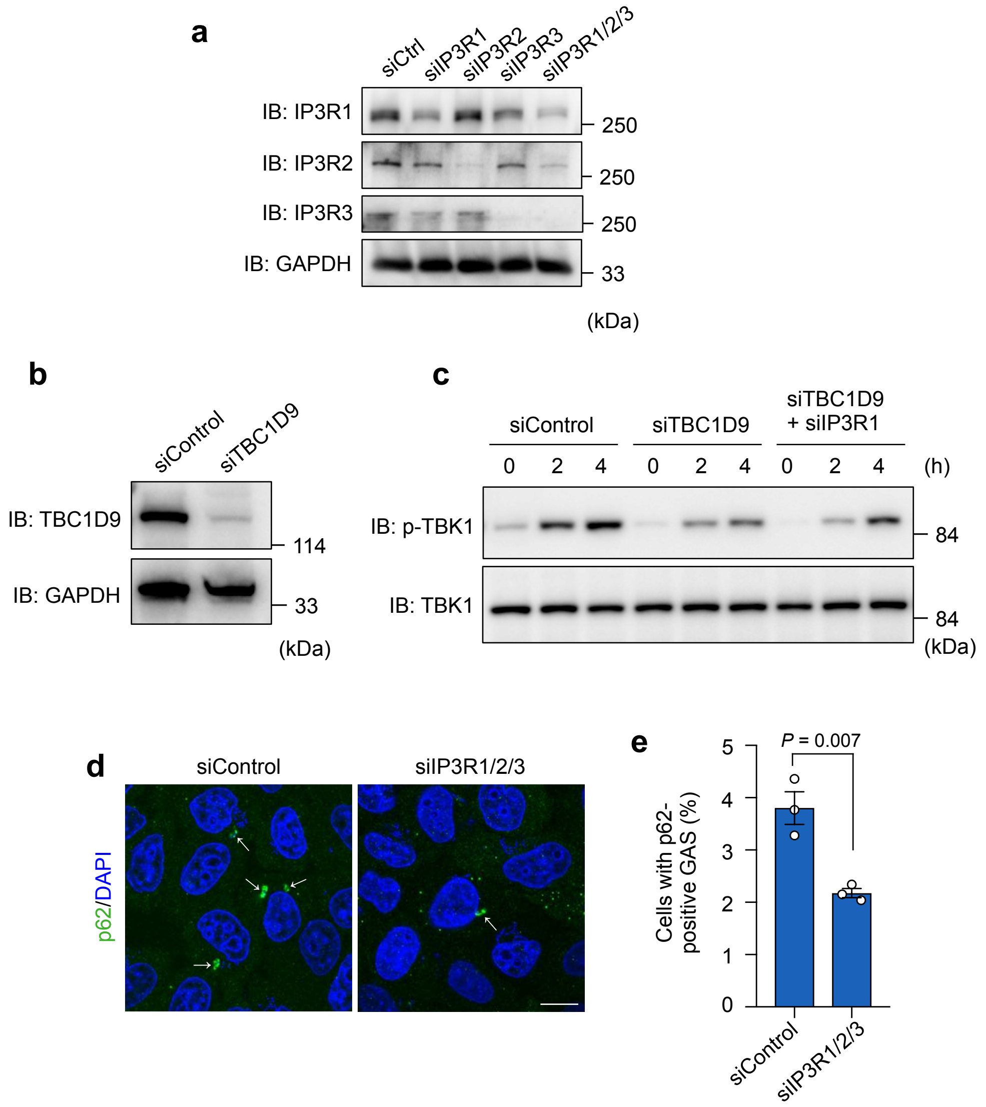

Supplementary Figure 7 IP3R is critical for TBK1 activation during GAS infection.
a Immunoblotting of siRNA-transfected cells. HeLa cells were transfected with indicated siRNA oligonucleotides for 48 h, and cell lysate were analyzed by immunoblotting using specific antibodies. **b, c** Effects of IP3R-depletion on the recruitment of p62 to GAS. HeLa cells transfected with indicated siRNAs were infected with GAS for 4 h, fixed, and stained for p62. **b** Representative confocal images and **c** the percentage of infected-cells showing p62-positive GAS. **d** Immnoblotting of *TBC1D9*-knockdown cells. **e** HeLa cells transfected with indicated siRNAs were infected with GAS for 4 h, and cell lysates were analyzed by immunoblotting using indicated antibodies. Scale bars, 10 μm. Data in **c** represent the mean ± SEM of three independent experiments. *P*-values calculated by two- tailed Student's t-test.

Supplementary Figure 8

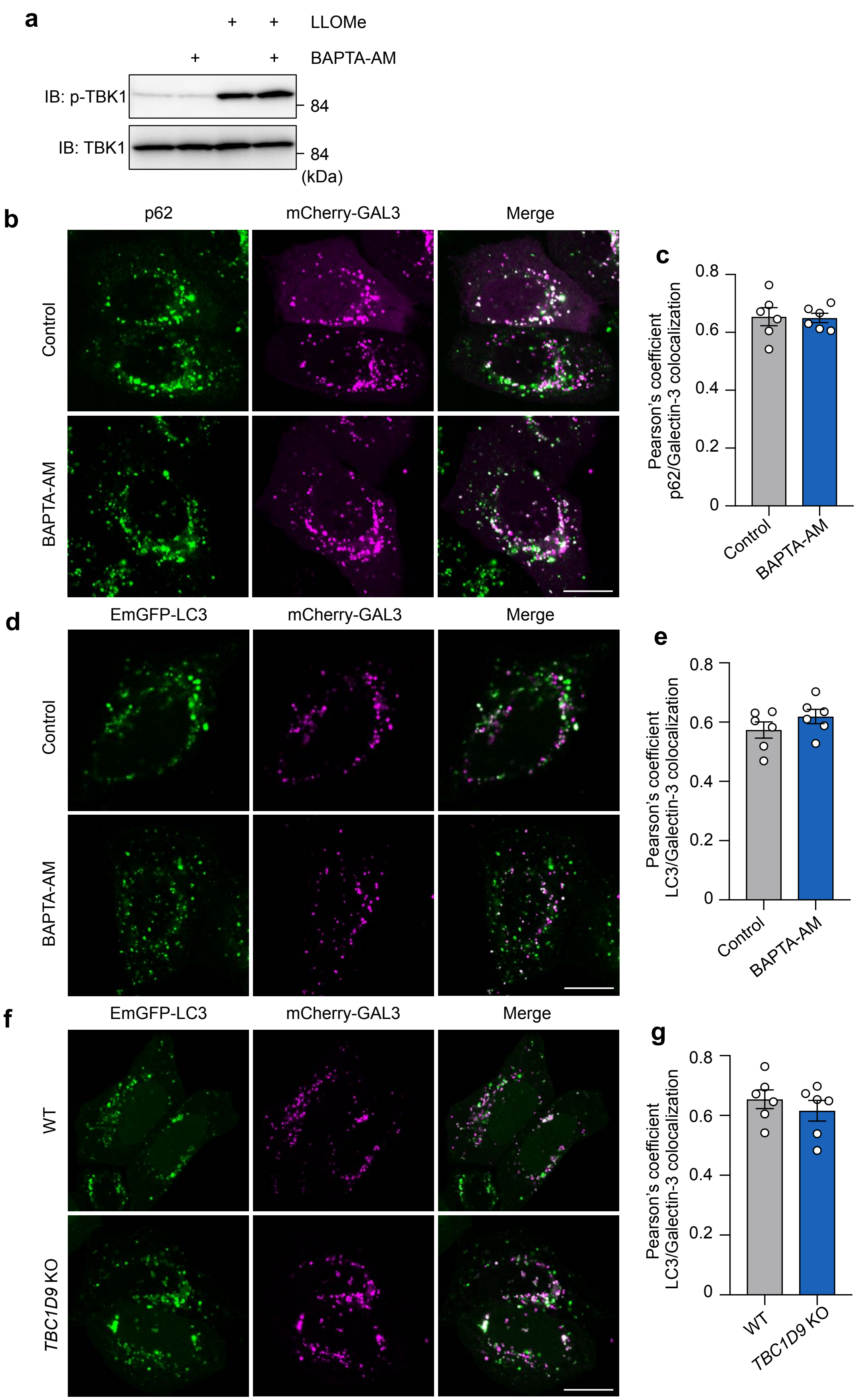

Supplementary Figure 8 Ca^{2+} and TBC1D9 are dispensable for lysophagy.
a HeLa cells treated with LLOMe and/or BAPTA-AM were analyzed by immunoblotting using indicated antibodies. **b,c** HeLa cells expressing mCherry-GAL-3 were treated with BAPTA-AM and LLOMe for 3 h. **b** Confocal images and **c** quantification of colocalization between p62 and GAL-3. **d,e** HeLa cells expressing EmGFP-LC3 and mCherry-Galectin-3 were treated with BAPTA-AM and LLOMe for 3 h. **d** Confocal images and **e** quantification of colocalization between LC3 and GAL-3. **f,g** HeLa WT and *TBC1D9*-KO cells expressing mCherry-GAL-3 were treated with LLOMe for 3 h. **f** Confocal images and **g** quantification of colocalization between LC3 and Galactin-3. Scale bars, 10 µm. Data in **c**, **e,** and **g** represent the mean ± SEM of three independent experiments. *P*-values calculated by two- tailed Student's t-test.

Supplementary Figure 9

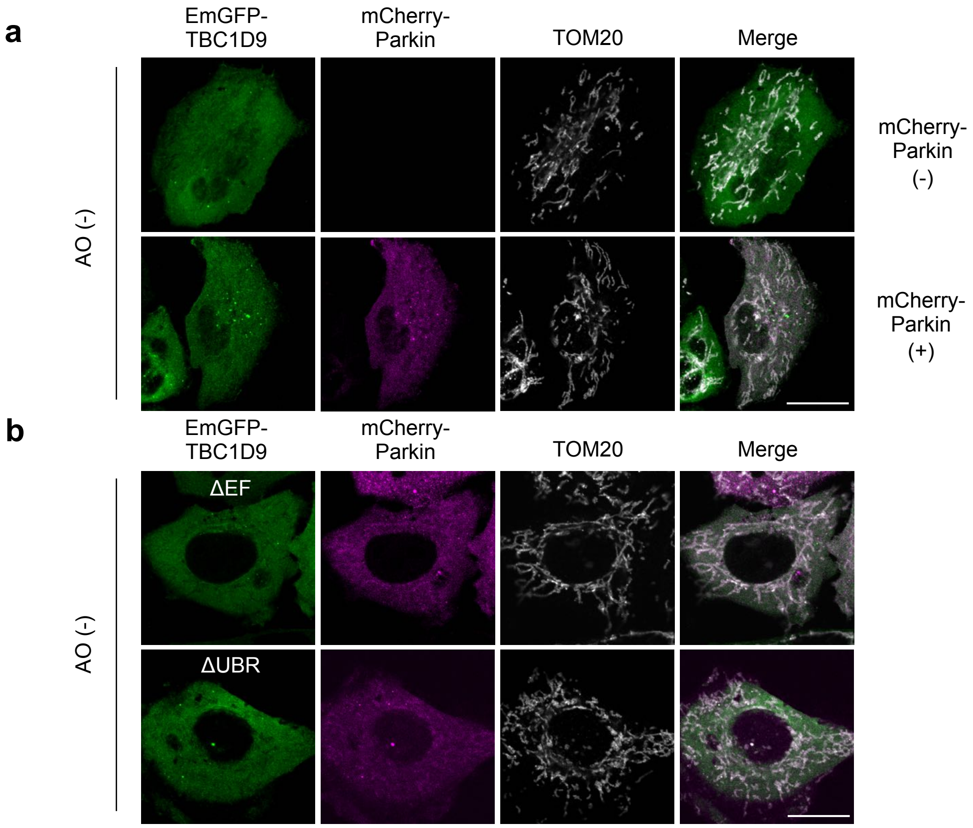

Supplementary Figure 9 Subcellular localizations of TBC1D9 and Parkin.
a HeLa cells expressing EmGFP-TBC1D9 and mCherry-Parkin were fixed and immunostained for TOM20. **b** HeLa cells expressing EmGFP-TBC1D9 ΔEF or ΔUBR and mCherry-Parkin were fixed and immunostained for TOM20. Scale bars, 10 μm.
