# Supplementary material for: TBC1D9 regulates TBK1 activation through Ca2+ signaling in selective autophagy
Source: Nat Commun. 2020 Feb 7;11:770. doi: 10.1038/s41467-020-14533-4 (PMC7005872; doi:10.1038/s41467-020-14533-4)
Supplement: Supplementary file 3 — Reporting Summary [file 41467_2020_14533_MOESM3_ESM.pdf]

nature research

Corresponding author(s): Ichiro Nakagawa
Last updated by author(s): Dec 17, 2019

Reporting Summary

Nature Research wishes to improve the reproducibility of the work that we publish. This form provides structure for consistency and transparency in reporting. For further information on Nature Research policies, see Authors & Referees and the Editorial Policy Checklist .

Statistics

For all statistical analyses, confirm that the following items are present in the figure legend, table legend, main text, or Methods section.

n/a	Confirmed	
☐	☒	The exact sample size (*n*) for each experimental group/condition, given as a discrete number and unit of measurement
☐	☒	A statement on whether measurements were taken from distinct samples or whether the same sample was measured repeatedly
☐	☒	The statistical test(s) used AND whether they are one- or two-sided<br>*Only common tests should be described solely by name; describe more complex techniques in the Methods section.*
☒	☐	A description of all covariates tested
☒	☐	A description of any assumptions or corrections, such as tests of normality and adjustment for multiple comparisons
☐	☒	A full description of the statistical parameters including central tendency (e.g. means) or other basic estimates (e.g. regression coefficient) AND variation (e.g. standard deviation) or associated estimates of uncertainty (e.g. confidence intervals)
☐	☒	For null hypothesis testing, the test statistic (e.g. *F*, *t*, *r*) with confidence intervals, effect sizes, degrees of freedom and *P* value noted<br>*Give P values as exact values whenever suitable.*
☒	☐	For Bayesian analysis, information on the choice of priors and Markov chain Monte Carlo settings
☒	☐	For hierarchical and complex designs, identification of the appropriate level for tests and full reporting of outcomes
☐	☒	Estimates of effect sizes (e.g. Cohen's *d*, Pearson's *r*), indicating how they were calculated

Our web collection on statistics for biologists contains articles on many of the points above.

Software and code

Policy information about availability of computer code

Data collection	Western blot densitometry data were collected by GE Healthcare ImageQuant LAS 4000 mini. Confocal microscopy data were acquired by Olympus FV1000.
Data analysis	Western blot data analysis was performed using ImageJ, confocal microscopy data analysis was done using Olympus FV1000 software or ImageJ 1.52 (NIH). All other data analyses were performed using GraphPad Prism 8 (GraphPad Software).

For manuscripts utilizing custom algorithms or software that are central to the research but not yet described in published literature, software must be made available to editors/reviewers. We strongly encourage code deposition in a community repository (e.g. GitHub). See the Nature Research guidelines for submitting code & software for further information.

Data

Policy information about availability of data

All manuscripts must include a data availability statement. This statement should provide the following information, where applicable:
- Accession codes, unique identifiers, or web links for publicly available datasets
- A list of figures that have associated raw data
- A description of any restrictions on data availability

All data that support the findings of this study are available from the corresponding author upon reasonable request.

Field-specific reporting

Please select the one below that is the best fit for your research. If you are not sure, read the appropriate sections before making your selection.

☒ Life sciences ☐ Behavioural & social sciences ☐ Ecological, evolutionary & environmental sciences

For a reference copy of the document with all sections, see nature.com/documents/nr-reporting-summary-flat.pdf

Life sciences study design

All studies must disclose on these points even when the disclosure is negative.

Sample size	No statistical method was used to predetermine sample size. Number of analyzed cells or measurements were chosen to equal or exceed typical standards of the field.
Data exclusions	No data was excluded.
Replication	All results were reproduced at least three times. All attempt for replication were successful and all experiments can be reproduced.
Randomization	Experiments were not randomized, but independent cultures or passages were used for each independent repeat and done on different days.
Blinding	Investigators were not blinded during data acquisition. Analysis of bands or fluorescence intensities was performed by software. But, investigation of the recruitment of proteins to specific targets such as bacteria or autophagosomes were manually counted.

Reporting for specific materials, systems and methods

We require information from authors about some types of materials, experimental systems and methods used in many studies. Here, indicate whether each material, system or method listed is relevant to your study. If you are not sure if a list item applies to your research, read the appropriate section before selecting a response.

Materials & experimental systems

n/a	Involved in the study	
☐	☒	Antibodies
☐	☒	Eukaryotic cell lines
☒	☐	Palaeontology
☒	☐	Animals and other organisms
☒	☐	Human research participants
☒	☐	Clinical data

Methods

n/a	Involved in the study	
☒	☐	ChIP-seq
☒	☐	Flow cytometry
☒	☐	MRI-based neuroimaging

Antibodies

Antibodies used

TBK1 (Abcam, ab40676, 1:1000 for WB,
p-TBK1 (Ser172) (CST, #5483, 1:1000 for WB, 1:100 for IF)
STING/TMEM173 (Proteintech, 19851-1-AP, 1:1000)
p-IRF3 (Ser396) (CST, #4947, 1:1000)
IRF3 (Santa Cruz, #33641, 1:500)
TBC1D9 (Bethyl Laboratories, #A301-028A; 1:1000 for WB, 1:100 for IF)
IP3R (CST, #3763, 1:1000)
ULK1 (CST, #6439, 1:1000)
ULK2 (GeneTex, #GTX111476, 1:500)
FIP200 (Proteintech, #17250-1-AP, 1:1000)
ATG13 (CST, #13468, 1:1000)
LC3B (Abcam, #ab51520, 1:1000)
β-actin (CST, #8457, 1:1000)
IP3R2 (Santa Cruz, sc-3988434, 1:500)
FLAG (Sigma, #A2220, 1:1000)
TOM20 (Santa Cruz, #sc-17764, 1:2000)
GAPDH (Santa Cruz, sc-32233, 1:1000)
COXII Abcam, #ab110258, 1:500)
GFP (Nacalai, #04363-24, 1:1000 for WB, 1:100 for IF)
p62 (Santa Cruz, #sc-28359, 1:100)
poly-ubiquitin (Nippon Bio-Test Laboratories, #MFK-004, 1:200)
p62 (Santa Cruz, #sc-25575, 1:100)
Lys63-specific ubiquitin (Millipore, #05-1308, 1:100)
Lys48-specific ubiquitin (Millipore, #05-1307, 1:100)
TOM20 (Santa Cruz, sc-17764, 1:100)
Peroxidase Goat Anti-Mouse IgG (H+L) (Jackson ImmunoResearch, #115-035-146, 1:5000)
Peroxidase Goat Anti-Rabbit IgG (H+L) (Jackson ImmunoResearch, #111-035-144, 1:5000)

	Anti-rabbit IgG (conformation specific) (HRP-Conjugate) (CST, #5127, 1:5000)<br>anti-Mouse IgG (H+L) Highly Cross-Adsorbed Secondary Antibody, Alexa Fluor Plus 488 (Invitrogen, #A32723, 1:500)<br>anti-Mouse IgG (H+L) Highly Cross-Adsorbed Secondary Antibody, Alexa Fluor Plus 594 (Invitrogen, #A32742 1:500)<br>anti-Rabbit IgG (H+L) Highly Cross-Adsorbed Secondary Antibody, Alexa Fluor Plus 488 (Invitrogen, #A32731, 1:500)<br>anti-Rabbit IgG (H+L) Highly Cross-Adsorbed Secondary Antibody, Alexa Fluor Plus 594 (Invitrogen, #A32740 1:500)
Validation	The antibodies that have been validated by the suppliers for specific proposes were purchased for our experiment.<br>In addition, for key antibodies in our study (TBK1, TBC1D9, IP3R), we validated the antibodies by western blotting upon depletion of the proteins.

Eukaryotic cell lines

Policy information about cell lines

Cell line source(s)	HeLa cell line was obtained directly from the American Type Culture Collection.
Authentication	Cell lines were not independently authenticated.
Mycoplasma contamination	Cell lines were routinely tested for mycoplasma and were certified to be negative
Commonly misidentified lines<br>(See ICLAC register)	No cell lines used in this study were found in the database of commonly misidentified cell lines.
