# Supplementary figures and images for: TBC1D9 regulates TBK1 activation through Ca2+ signaling in selective autophagy
Source: Nat Commun. 2020 Feb 7;11:770. doi: 10.1038/s41467-020-14533-4 (PMC7005872; doi:10.1038/s41467-020-14533-4)

Figure 1

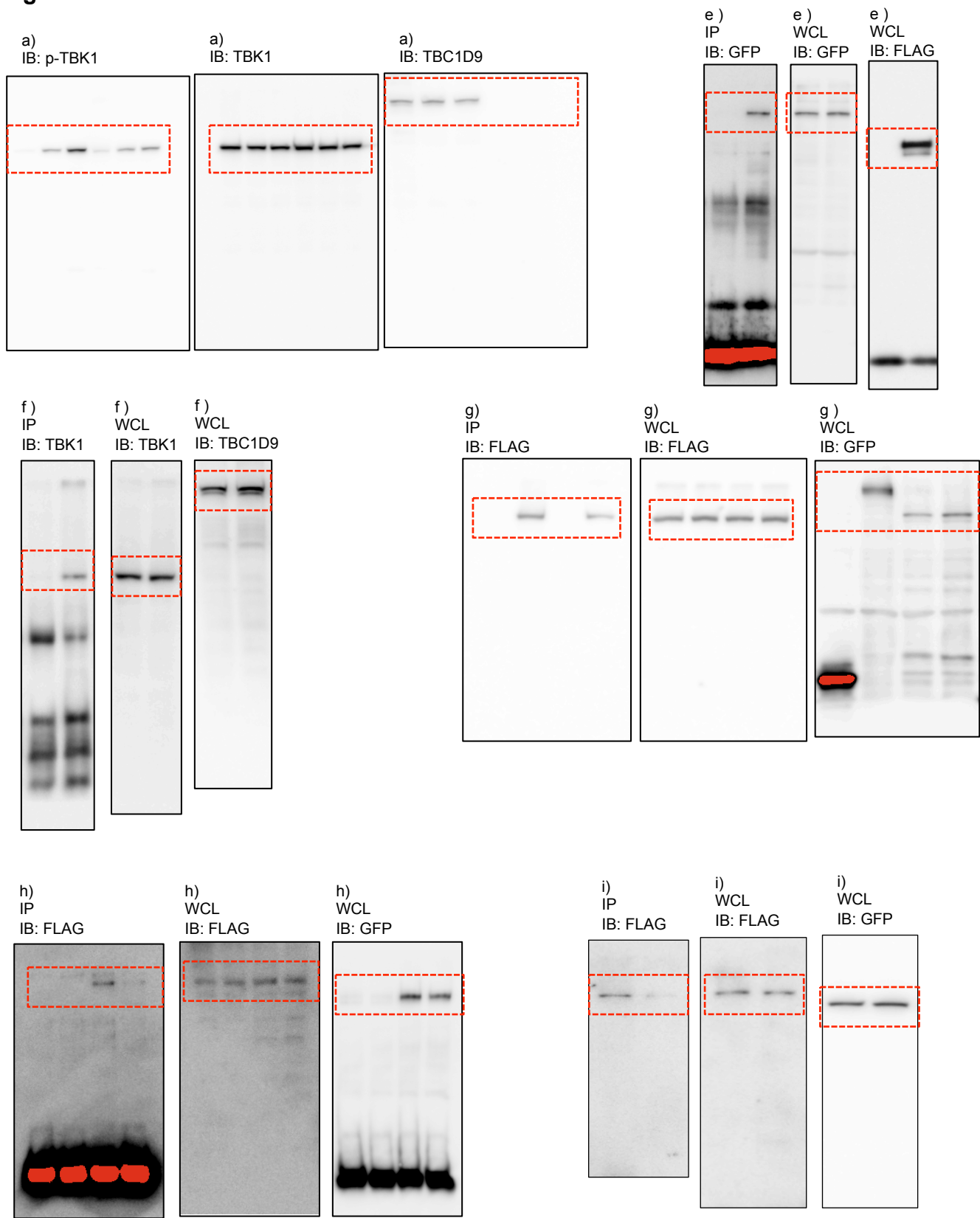

Figure 2

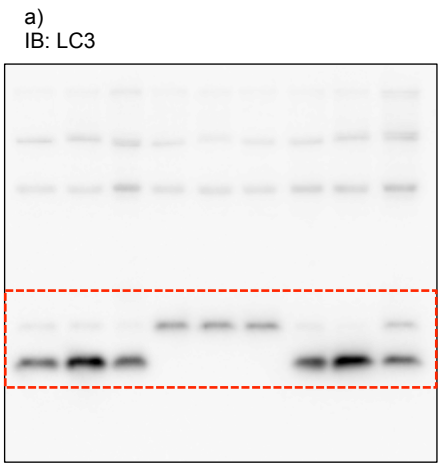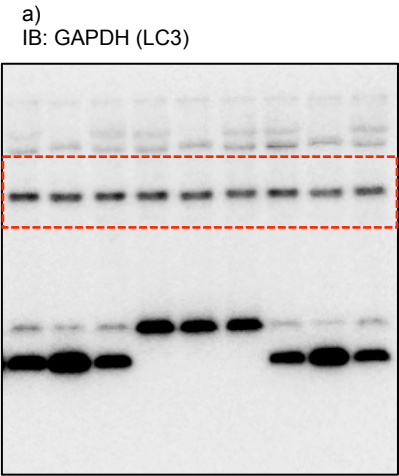

Figure 3

g)  
IB: GFP

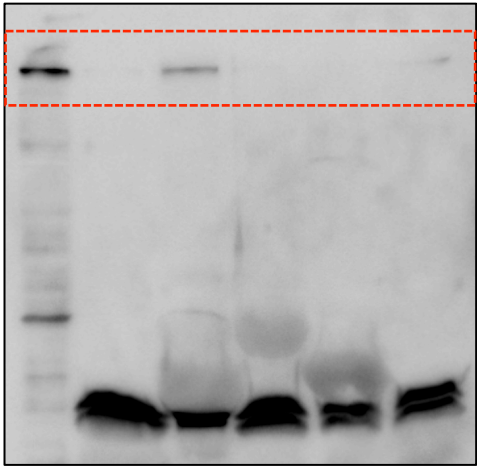

g)  
CBB staining

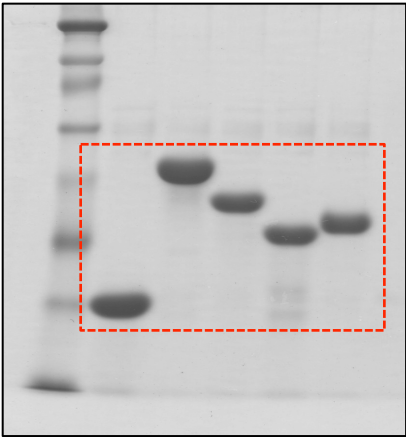

h)  
IB: ubiquitin

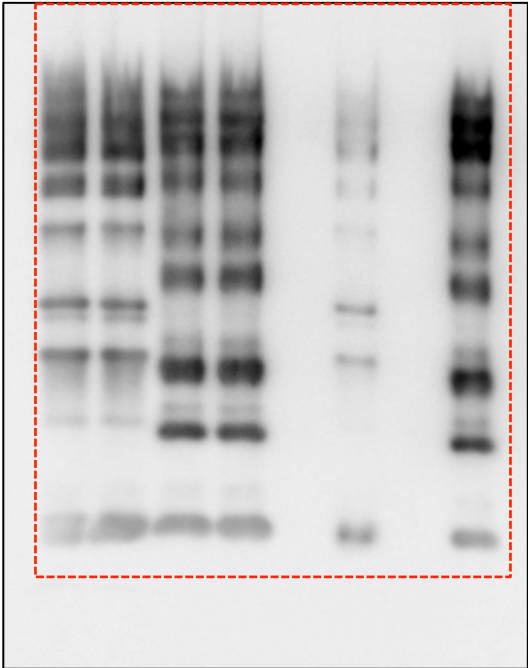

h)  
IB: GST

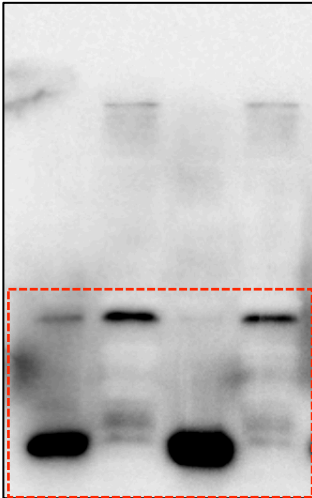

Figure 4

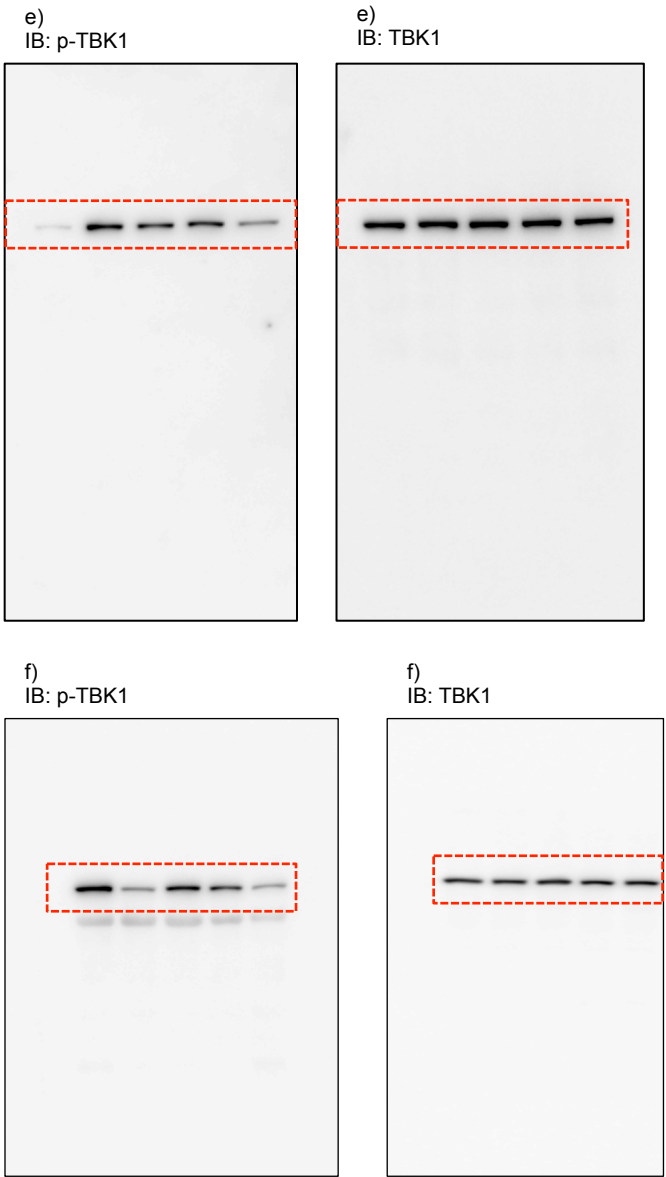

Figure 6

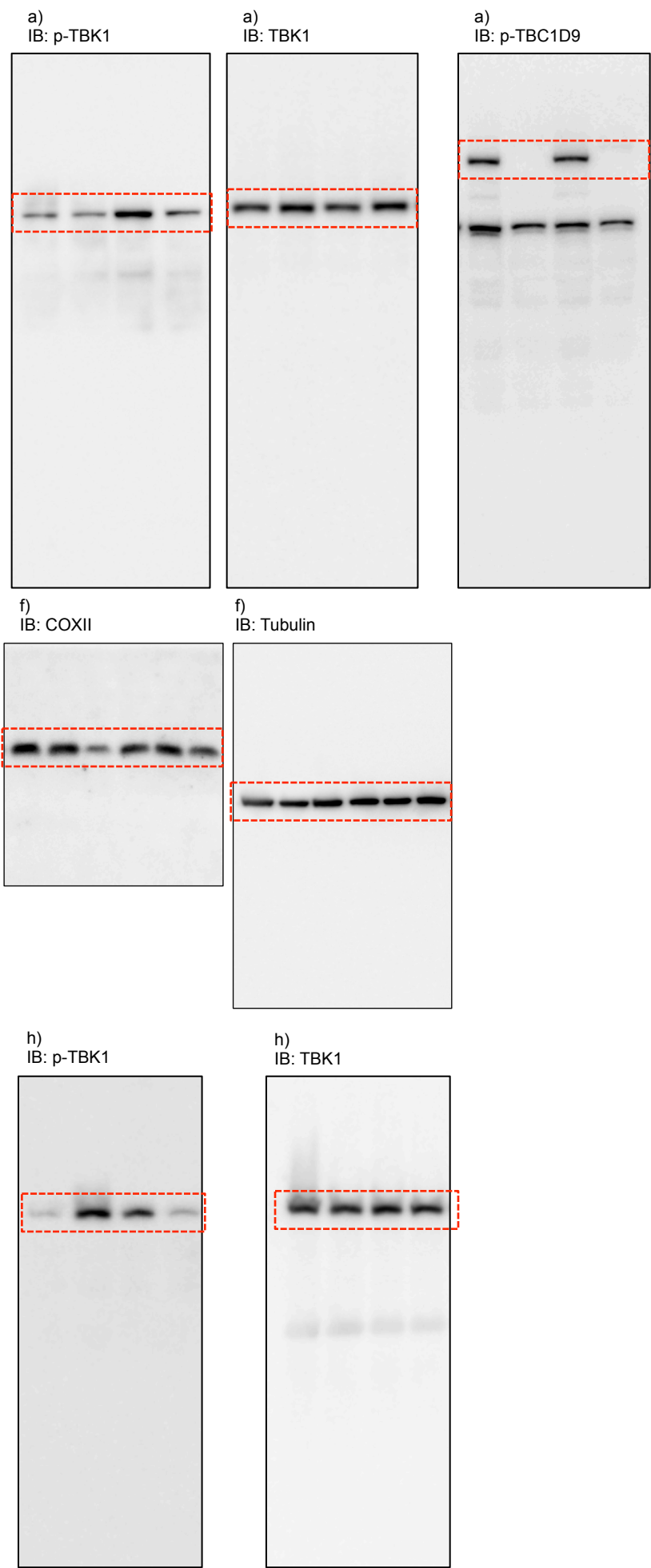

Supplement: Supplementary file 4 — Source Data [file 41467_2020_14533_MOESM4_ESM.zip › 1.pdf]
